# Three-Dimensional Deep Learning with Routine Brain Magnetic Resonance Imaging and Clinical Data for Identification of Secondary Progressive Multiple Sclerosis

**DOI:** 10.3390/brainsci16070670

**Published:** 2026-06-26

**Authors:** Mahshid Soleymani, Olayinka Oladosu, Saahim Salman, Mahum Rashid, Mariana Bento, Yunyan Zhang

**Affiliations:** 1 Department of Biomedical Engineering, University of Calgary, Calgary, AB T2N 4N1, Canada; 2 Department of Radiology, University of Calgary, Calgary, AB T2N 4N1, Canada; 3 Hotchkiss Brain Institute, Cumming School of Medicine, University of Calgary, Calgary, AB T2N 4N1, Canada; 4 Department of Clinical Neurosciences, University of Calgary, Calgary, AB T2N 4N1, Canada

**Keywords:** classification, clinical brain MRI, 3D deep learning, 3D Grad-CAM, phenotype, multiple sclerosis

## Abstract

**Highlights:**

**What are the main findings?**

- Three-dimensional deep learning models such as VGG19 have the potential to distinguish individuals with SPMS from RRMS using routine brain MRI and clinical data.
- Explanation studies of the VGG19 model assisted by a three-dimensional Grad-CAM method indicated brain regions of significance in distinguishing SPMS from RRMS including bilateral frontal lobes, left occipital and temporal lobes, and cerebellum.

**What are the implications of the main findings?**

- Three-dimensional deep learning along with model explanation could facilitate early accurate identification of individuals with SPMS and discovery of new biomarkers underlying disease worsening in MS.

**Abstract:**

**Objectives**: Secondary progressive multiple sclerosis (SPMS) is a natural transition from relapsing-remitting multiple sclerosis (RRMS) in many cases. However, whether and how these phenotypes differ on an individual basis is not fully understood, limiting timely diagnosis and management for SPMS. This study aimed to investigate how deep learning using 3-dimensional (3D) frameworks including VGG19, ResNet152, and DenseNet-121 helped differentiate SPMS from RRMS based on routine clinical datasets, and what brain areas mostly contributed to this differentiation using model explanation techniques. **Methods**: We examined 140 participants (70 each for RRMS and SPMS) as part of an ongoing study comprising prospectively collected clinical and imaging data from routine healthcare. The data was curated to improve consistency and completeness using different strategies and were then randomly split by subject into training (n = 120) and held-out testing (n = 20). The former was used for model development through five-fold cross validation. Deep learning used T1-weighted, T2-weighted, and FLAIR brain MRI, with optional clinical variables (n = 6). A 3D gradient-weighted class activation mapping (Grad-CAM) technique was applied to identify brain areas of significance followed by ablation studies for additional insight. **Results**: Among the 3D frameworks validated, VGG19 was deemed the best. Based on MRI and the best 3D VGG19 model, different data curation strategies showed largely similar results. Additionally, the models combining clinical variables with MRI achieved equivalent or slightly greater performance than MRI-only models, with an average testing area under the receiver operating characteristic curve of 0.84 when datasets were fused at the flatten layer, best at 0.92, versus 0.82 and 0.89. Model explanation indicated brain regions of significance in distinguishing SPMS from RRMS individuals, including bilateral frontal lobes, left occipital and temporal lobes, and cerebellum. **Conclusions**: Overall findings suggest the potential of 3D deep learning models such as VGG19 for distinguishing SPMS from RRMS using routine brain MRI and clinical data, which, along with 3D Grad-CAM, could facilitate discovery of new biomarkers underlying disease worsening.

## 1. Introduction

Multiple sclerosis (MS) is a prevalent and highly heterogeneous disease of the central nervous system characterized by inflammation, demyelination, and degeneration [1]. Most people with MS begin with a relapsing-remitting phenotype (RRMS) [2], yet over 50% of them worsen to secondary progressive MS (SPMS) within 10 to 15 years of onset without appropriate treatment [3]. The transition from RRMS to SPMS marks a dramatic change in management strategy. However, timely diagnosis of SPMS remains a considerable challenge for individuals [4,5], because each individual has different disease characteristics [6]. The development of new approaches such as deep learning to discern SPMS from RRMS is desperately needed for precision care.

Deep learning techniques are ideal candidates for characterizing individualized disease outcomes as represented by convolutional neural networks (CNNs) [7,8]. These methods can automatically extract patterns and trends concealed in existing datasets and then use the learned knowledge to predict outcomes for a new individual. Previously, a study using 2-dimensional (2D) CNN models showed over 90% accuracy in differentiating MS from healthy control subjects [9]. Using 3D CNNs that permitted volumetric feature extraction, recent work also demonstrated the potential to distinguish sub-cohorts of MS. Specifically, with a 3D Visual Geometry Group (VGG) approach [10], one study achieved 84% accuracy in classifying primary progressive MS vs. RRMS based on T1-weighted brain MRI of 91 subjects. Using a 3D ResNet CNN on T1-weighted and fluid-attenuated inversion recovery (FLAIR) brain MRI of 319 subjects [11], another study obtained testing accuracies of 71% to 79% in classifying early MS into subsets of high or low expanded disability status scale (EDSS). However, identifying SPMS vs. RRMS with 3D deep learning methods is limited. In addition, there was little evidence in the literature on clinically standard MRI that was known to be more heterogeneous and therefore challenging to handle than research-quality data [12].

The goal of this study was to develop a 3D deep learning model for differentiation of individuals with SPMS vs. RRMS based on routine clinical datasets especially brain MRI. Simultaneously, another purpose was to explore which brain areas contributed the most to this detection using a 3D model explanation technique termed gradient-weighted class activation mapping (Grad-CAM) [13,14].

## 2. Materials and Methods

### 2.1. Dataset

This research investigated 140 participants (70 each with RRMS and SPMS) involved in an ongoing study on the Clinical Impact of MS (CIMS, REB14-1926). The CIMS database contained prospectively collected clinical and imaging data collected from routine healthcare since 1996. Inclusion criteria for the present study were: (1) a diagnosis of RRMS or SPMS; (2) presence of at least one brain MRI scan and associated clinical measures (up to 2019); and (3) no other neurological disorders. For technical reasons, only scans acquired in 2002 or later were usable and therefore investigated. Clinical variables commonly associated with MS activity included: age at disease onset and at the time of scan, sex, EDSS score at onset and at the time of scan, disease duration, and treatment duration at the time of scan (Table 1). The latter followed regular clinical care practices using approved disease modifying therapies including glatiramer acetate, interferon-beta, teriflunomide, ocrelizumab, and dimethyl fumarate. This research adheres to the Declaration of Helsinki and it was approved by the institutional ethics review board. All participants provided written informed consent.

### 2.2. Imaging Protocol

To understand clinical relevance, we focused on typical MS imaging protocols including T1-weighted (T1), T2-weighted (T2), and FLAIR brain MRI. The imaging was conducted with five different scanners from Siemens (Erlangen, Freistaat, Germany) and GE Medical corporations (Milwaukee, WA, USA) at 1.5 T or 3 T, with different acquisition settings. Specifically, T1 MRI mostly used a 3D fast spoiled or magnetization-prepared rapid gradient-echo sequence, with a repetition time (TR) = 6.9 to 2300.0 ms, echo time (TE) = 2.3 to 19.9 ms, matrix size = 256 × 192 to 512 × 512, and slice thickness = 1 mm to 5.5 mm. T2 MRI used primarily a fast spin-echo sequence, with TR = 2975.0 to 11,009.9 ms, TE = 82.5 to 122.7 ms, matrix size = 256 × 256 to 512 × 512, and slice thickness = 1 mm to 5.5 mm. Representative FLAIR sequences were T2-weighted or 3D Turbo spin echo, with TR = 5000.0 to 9475.0 ms, TE = 93.0 to 388.0, matrix size = 256 × 200 to 512 × 512, and slice thickness = 0.5 mm to 5 mm. Depending on clinical needs, not all participants had all three sequences obtained.

### 2.3. Image Preparation

Several steps were conducted to improve image quality and consistency. The scans were isotropic-adjusted and then skull stripped using the FSL tool (FMRIB, Oxford, UK), followed by bias field correction with the N4 algorithm implemented in the ANTsPy library [15]. The next step was co-registration, from local T1 to the standard MNI152 1 mm T1 template as well as from local T2 and FLAIR to local T1 and then to the MNI-aligned T1. The last step was image intensity normalization, done with a ‘Z-score’ method based on this formula: Brain_norm (i)=(Brain(i)−mean(Brain))/SD(Brain). Brain(i) referred to the *i*th voxel intensity of a scan, and SD(Brain) was standard deviation of voxels per sequence per participant.

Another associated procedure was dealing with unavailable sequences as commonly seen in clinical imaging. For scans with missing T1, we experimented with two approaches to compare. The first was to replace the images with a customized T1-template. The template was achieved based on T1 sequences that had the same dimensions in a group such as RRMS or SPMS. As these available T1 sequences had already been aligned with the same MNI T1 template, they were averaged to provide the template. Using the replacement T1, co-registrations were then done as noted above for involved subjects. The second was to generate subject-wise T1 images from corresponding FLAIR (or T2 if FLAIR was not ideal) using our pre-trained models based on the cycle-consistent generative adversarial network (CycleGAN) [16,17]. The CycleGAN was a recognized deep learning approach for conducting individualized image translation that was used frequently in medical imaging. In cases where T2 or FLAIR was unavailable, it was substituted with each other. In scans where both T2 and FLAIR were missing, the T1 scan was used instead.

Finally all axial brain MRI slices were zero-padded to generate square-like dimensions of 182 × 256 × 256. To reduce computational burden, the scans were 2× down sampled to 91 × 128 × 128 per MRI sequence for deep learning.

### 2.4. Clinical Data Curation

Clinical variables were also curated to increase consistency for effective deep learning when they were investigated together with MRI data. This process was done based on variable type. Age and disease duration were each ordinal encoded to a 100 × 1 vector as endorsed by prior work [18]. This approach was taken to extend the representation of these features because otherwise they would appear minimal as compared to imaging features. The 100 × 1 vector was composed of ones with duration of the “variable value”, such as 25 ones for an age of 25 years old, and zeros over the rest 75 (100 minus variable value) cells. To compare, we also took a different curation approach where the age variables were grouped and coded into five categories as ~15 to <20, 20 to <25, 25 to <35, 35 to <50, and 50 to <75. The disease duration years were not binned as the range was not as wide as age. The sex and EDSS scores were converted to categorical vectors, with sex becoming a binary vector corresponding to female and male. The EDSS scores in this study ranged between 0 and 8.0 at 0.5 increases and therefore were converted to integers of length 17 × 1. Each participant score was then represented by an one-hot encoded vector.

### 2.5. Model Development

**Figure 1:**
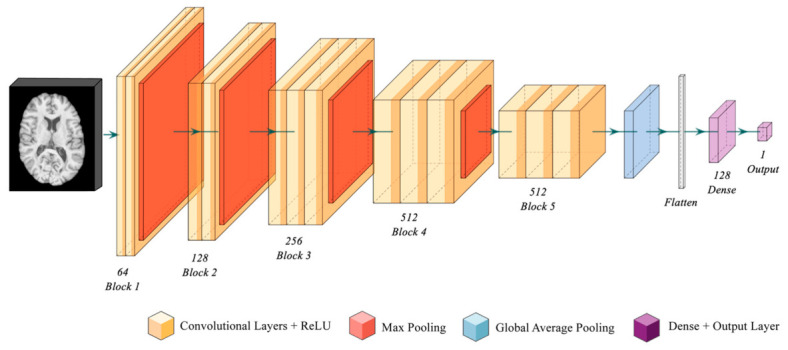
The 3-dimensional (3D) VGG19 model architecture. The images represent 3D brain MRI data used as input to the network. The numbers under color diagrams indicate the number of filters (used in feature extraction with individual 3D convolutional blocks), and number of nodes (applied with fully connected or dense layers).

Deep learning used 3D models through transfer learning based on recognized CNN methods especially VGG19 [19], which was compared with the 3D ResNet152 [20] and 3D DenseNet-121 [21]. All were pretrained on large ImageNet datasets [22]. Transfer learning was shown to be effective in handling relatively small sample sizes [23]. The VGG19 model consisted of five 3D convolutional blocks for feature extraction, followed by global average pooling, flatten, and fully connected layers, culminating in a binary output layer (SPMS or not) activated by sigmoid nonlinearity (Figure 1). The 3D ResNet152 model was also a sequential method composed of 152 layers, with the unique residual connections included to resolve the vanishing gradient problem for efficient learning. This particular model version was chosen because it had a similar number of parameters to 3D VGG19. Unlike sequential models, the 3D DenseNet-121 connects every layer to every subsequent layer of the network by concatenation of features. In this way, information from earlier layers was effectively propagated to later layers for optimal learning.

Based on the best MRI only model found above, we also investigated how deep learning worked using combined MRI and clinical variable datasets (Combined model). In these experiments, the variables were fused with MRI features for joint decision making using either of the three approaches for comparison: (1) at the flatten layer; (2) at the first fully connected layer; and (3) at the second fully connected layer (temporarily added for this testing purposes, 64 nodes). Notably, the EDSS scores at scan were presumably highly distinct between the RRMS and SPMS groups given their inherent differences in clinical presentation, and therefore this variable was not directly tested with the Combined model to avoid bias.

**Figure 2:**
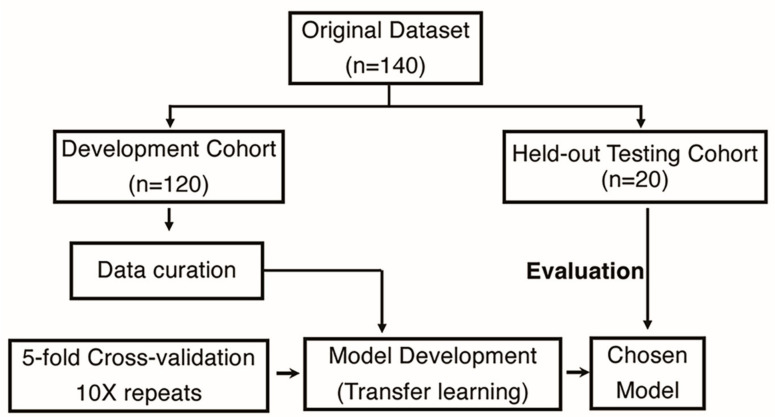
Data processing and analysis pipeline.

All 3D models were developed through 5-fold cross-validation using 86% of the data (120 subjects; 60 each for RRMS and SPMS) randomly split from the dataset. The remaining 14% of the data (10 subjects each for RRMS and SPMS) were used for held-out testing (Figure 2). All data splits were done at a subject level to avoid information overlap between portions. Additionally, the cross-validation experiments were repeated 10 times, each initiated with a random seed of 42 for stability.

Over training, the hyperparameters were set based on common practices as well as grid search findings. These included a learning rate scheduler with a starting point of 1 × 10^−4^ and a decay factor of 0.3, a batch size of eight, 30 epochs, and the Adam optimizer to minimize binary cross entropy as the loss function [24]. Early stopping was implemented with a patience of five by monitoring validation loss. The model with the lowest validation loss was deemed the best.

### 2.6. Model Testing

Several metrics were applied in model assessment using the held-out testing dataset, including accuracy, F1-score, precision, recall, and area under the receiver operating characteristic curve (AUC). Given our interests of properly identifying SPMS individuals, our true positive (TP) and true negative (TN) values reflected the numbers of correctly classified SPMS and RRMS, respectively. For the best models chosen, both mean and best overall performance metrics were reported, with the former derived by averaging values across 10 repeats. For side-by-side comparison experiments, mean AUC values were used primarily.

### 2.7. Model Explanation with 3D Grad-CAM

The best evaluation model was explored with a 3D Grad-CAM technique obtained from the Keras website [25]. This technique worked by leveraging gradients of the output score with respect to feature maps from the final convolutional layer, which created a localizing heatmap, $L_{Grad\text{-}CAM}^{c}$. It was defined as:

$$L_{Grad\text{-}CAM}^{c}=ReLU(\sum_{k}w_{k}^{c}A^{k})$$

Here $A^{k}$ was the k-th feature map of the final convolutional layer, and $w_{k}^{c}$ referred to weights (importance) of a feature map $A^{k}$ and was computed as an average of the gradients over spatial dimensions of $A^{k}$ The rectified linear unit (ReLU) function ensured that only positive values were considered. 

SPMS and RRMS participants who were correctly classified in the testing cohort were assessed. Grad-CAM values ≥95th percentile (95%ile) in a subject scan were included to focus on the most important voxels. Subsequently, voxel-wise one-sample and two-sample *t*-tests were applied, followed by correction for multi-voxel comparisons, which allowed identification of common brain areas within a group and between groups, respectively, as reported previously [14]. Specifically, in one-sample analyses, the Grad-CAM values were compared to a population mean, which was set to zero. For two-sample analyses, the values were compared between RRMS and SPMS cohorts. Ultimately, brain voxels with significant *t*-values were retained.

To understand the validity of identified brain regions, we also performed ablation studies. Essentially, based on the same testing dataset, we applied an established brain atlas to segment individual brains into different anatomical regions in MRI [26]. By masking out key brain regions including a whole left or right hemisphere, one region at a time, the chosen deep learning model was tested again per subject, with corresponding AUCs documented. Finally, a ‘significance score’ of a region was computed as percentage differences in AUCs based on brain areas without the region versus the whole brain area.

### 2.8. Statistical Analysis

Comparison of model performance used the Delong’s test for AUCs [27], where *p* ≤ 0.05 was considered significance. When identifying the most important brain areas for model decisions with 3D Grad-CAM, *p* < 0.00001 was considered significant in one-sample and two-sample *t*-tests, respectively, after correcting for multi-voxel comparisons. All statistical analyses were done using the R software.

## 3. Results

### 3.1. Data Characteristics

The SPMS group was older at the time of scan (*p* = 0.005), had more males (*p* = 0.005), and greater EDSS scores at onset and at the time of scan (*p* < 0.001) than in the RRMS group (see Table 1). Most of the SPMS participants had only recently transitioned to the progressive phase (mean = one year; range zero–10 years). The mean treatment duration at the time of scan was slightly shorter for RRMS participants than for SPMS participants (*p* = 0.050). Of the 140 participants, 29 in RRMS and 35 in SPMS had complete sequences. Initially T1 was unavailable the most (46% in RRMS and 30% in SPMS), followed by FLAIR and T2 (19% and 31%, and 13% and 11% in RRMS and SPMS). All of the involved scans were curated using respective methods as described above.

### 3.2. Model Performance from Different Architectures

Based on the same MRI only dataset where unavailable T1 images were curated using our customized template approach, all three deep learning methods achieved competitive results. Comparably, the mean (standard deviation) validation AUC was slightly higher with 3D DenseNet-121 and 3D VGG19 than 3D ResNet152 at 0.88 (0.08) and 0.85 (0.11), respectively, versus 0.77 (0.10). Regarding the best folds, the validation AUC of VGG19 was the best at 1.0, followed by DenseNet-121 at 0.97 and then ResNet152 at 0.89. In addition, the validation and training loss converged the best with the best VGG19 fold, followed by the best ResNet152 and then DenseNet-121 folds (Supplementary Figure S1). Accordingly, the best model from the 3D VGG19 method was used for subsequent experiments.

### 3.3. Model Performance Based on Different Image Augmentation Approaches

**Figure 3:**
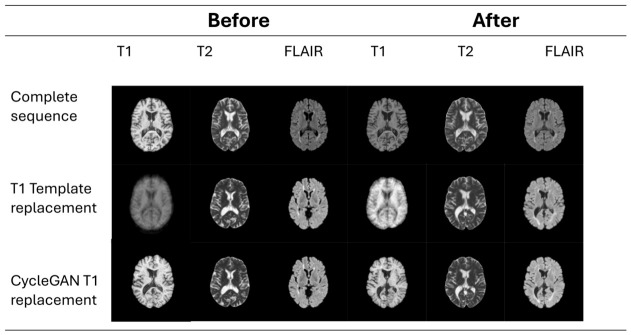
Example imaging co-registration schemes for subjects with or without ‘missing’ T1-weighted (T1) MRI. Top row shows a subject with all sequences obtained, so the images are co-registered to local T1 and then to the MNI T1 template. Middle row shows a subject without T1 acquired. It is first replaced by the T1 template generated in this study, which is then used to co-register local images to the MNI T1 template. Similarly, the bottom row also shows a subject without T1 acquired, while it is replaced by T1 images generated by the corresponding FLAIR using the cycle-consistent generative adversarial network (CycleGAN).

**Figure 4:**
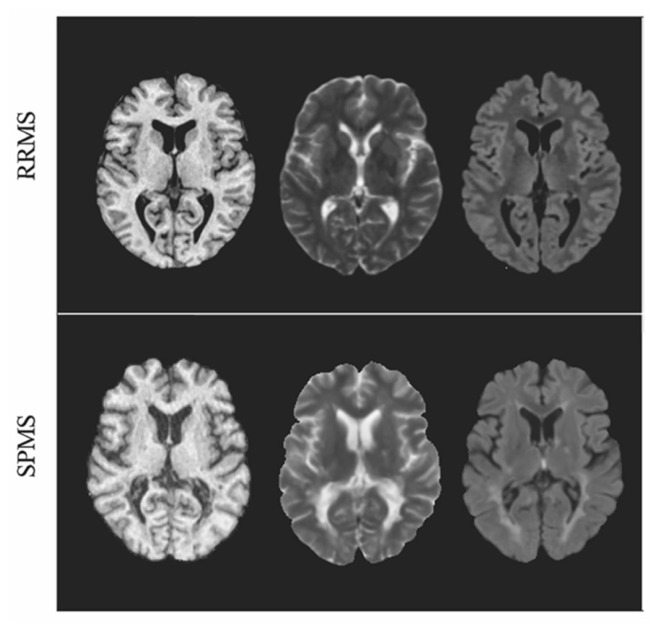
Example brain MRI slices from two correctly classified subjects. Shown from left to right are T1-weighted, T2-weighted, and FLAIR MRI. The RRMS subject was a female, with an onset age at 40s (50s years old at scan), EDSS score of 1.0, and disease duration of 13 years at scan. The SPMS subject was a male, with an onset age at 30s (40s years at scan), EDSS score of 3.0, and disease duration of 14 years.

Both our T1 template and CycleGAN methods provided T1 images that demonstrated high anatomical consistency with their corresponding T2 and FLAIR images. Following co-registration, all of the images were aligned with the same MNI T1 template (Figure 3). When classifying individuals with SPMS vs. RRMS conducted with only MRI data, the scans containing a T1 template or CycleGAN T1 images led to similar results based on the best VGG19 model identified above (Figure 4). Both showed the same average testing AUC at 0.82, while the other metrics appeared slightly higher with T1 template images than CycleGAN T1 images (Table 2). In all experiments, the same T2 and FLAIR images were applied.

### 3.4. Performance of Combined Model from Different Settings

**Figure 5:**
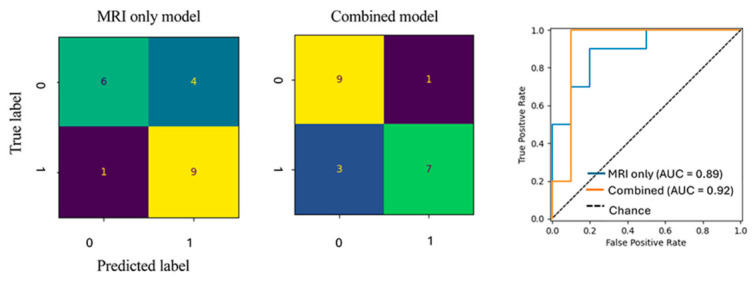
Best testing performance of the MRI-only model and MRI plus clinical variables (Combined) model. The zeros and ones in confusion matrices indicate relapsing-remitting multiple sclerosis and secondary progressive multiple sclerosis, respectively. Note: AUC refers to area under the receiver operating characteristic curve (AUC).

Depending on the data curation and fusion strategies applied, the Combined model performed slightly differently (see Table 2). Overall, when age variables were prepared in 100-dimension vectors (100 bins), the average testing performance of the models was consistently better when clinical variables were fused at the second fully connected layer than other fusion options. Conversely, when the variables were encoded as five bins, the Combined model showed mixed performance and most of its metrics appeared to be slightly better than models using 100 bins. Additionally with five bins, the mean testing AUC of combined models was similar to or slightly better than the MRI only model (*p* > 0.05), best at 0.84 as shown with data fusion at the flatten layer, where the best testing AUC was 0.92 (Figure 5).

### 3.5. Grad-CAM Along with Ablation Outcomes

Based on one-sample statistics, the frontal lobe formed the main region of significant, followed by partial parietal and occipital lobes in the RRMS group. In SPMS, the significant areas included additional parietal and occipital lobes, along with considerable areas of the frontal lobe, and partial temporal lobe and cerebellum.

**Figure 6:**
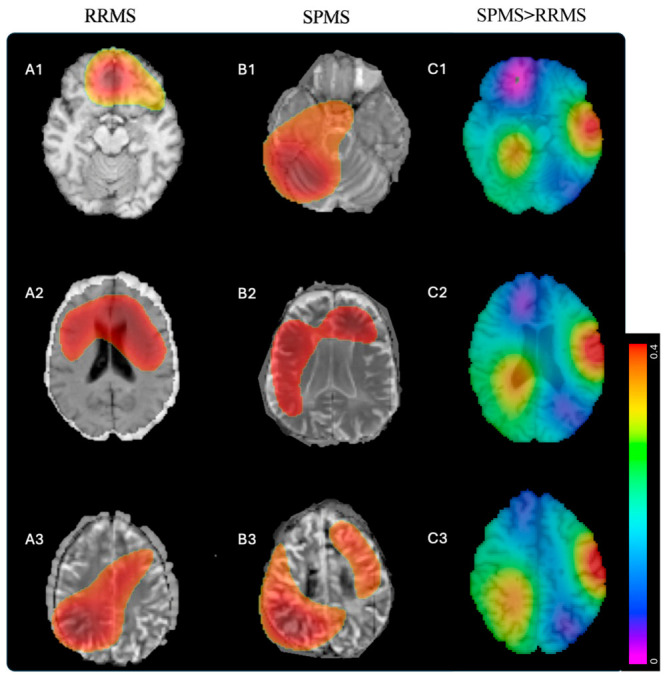
Example heatmaps of 3D gradient-weighted class activation mapping (Grad-CAM) overlaid on brain MRI. Different image slices from a testing subject are shown from relapsing-remitting multiple sclerosis (RRMS, (**A1**–**A3**)), involving mostly frontal and parietal lobes; a subject with secondary progressive multiple sclerosis (SPMS, (**B1**–**B3**)), involving temporal, frontal/parietal, and occipital lobes, as well as cerebellum; and group-based heatmaps in SPMS versus RRMS (**C1**–**C3**) as partially shown for the frontal/temporal and parietal/occipital lobes, and cerebellum.

From the two-sample statistics, more significant brain areas (higher *t*-values) were noted in the SPMS group than in RRMS group. These were noted mainly in the frontal/temporal lobes, occipital lobe, and then parietal lobe, as well as the cerebellum as highlighted in different brain MRI slices (Figure 6).

**Figure 7:**
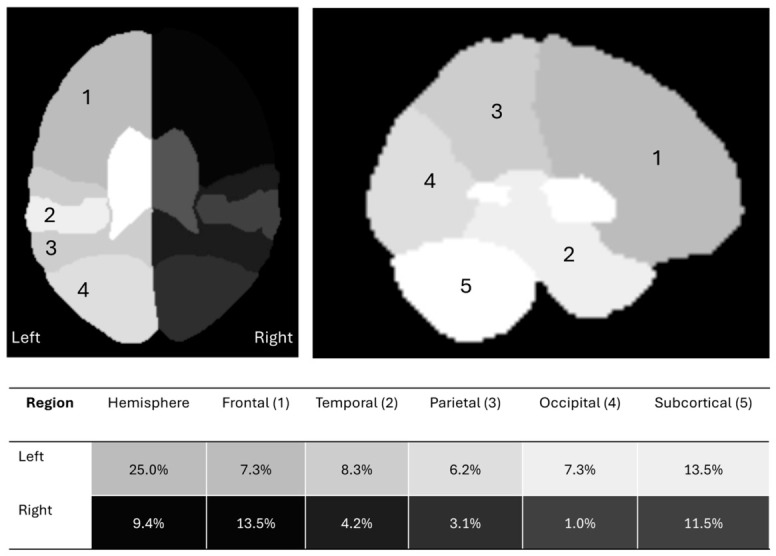
An atlas of the human cerebral lobes in MNI305 coordinates. The numbers represent different brain regions segmented corresponding to individual cerebral lobes and the cerebellum, where the latter is labeled as part of the subcortical region (number 5). This is the atlas used in a published article [26]. Gray shades in the Table indicate degrees of relevance, the darker the more relevance.

Based on ablation studies, different brain regions showed different significance in classifying subjects with SPMS from RRMS (Figure 7). Among regions explored in the testing set, the significance score was the highest in the right (radiological left) frontal lobe and cerebellum, both at 13.5%. This was followed by the left occipital lobe (8.3%), then left frontal and temporal lobes (7.3%), and then left parietal lobe (6.2%). Right brain regions except the frontal lobe showed lower scores than left regions. Furthermore, the left hemisphere showed a higher score at 25.0% than right hemisphere at 9.4%.

## 4. Discussion

Based on widely available clinical datasets, this study investigated the feasibility of identifying individuals with SPMS from RRMS using 3D deep learning techniques. Overall, the 3D VGG19 model appeared to be better than 3D ResNet152 and 3D DenseNet-121 models based on validation using MRI only data. Further analysis using the best VGG19 model showed that replacement of missing T1 using either customized T1 template or subject-wise CycleGAN images would not make a significant difference. The same applied to data curation options for clinical variables when they were tested in the Combined model along with MRI, although encoding continuous variables such as age into five bins appeared to be slightly better than 100 bins with data fusion at the flatten layer. Furthermore, 3D Grad-CAM along with ablation explorations suggested that several brain regions contributed significantly to the identification of SPMS from RRMS including the frontal lobes and cerebellum, as well as left occipital, temporal, and parietal lobes.

SPMS is a natural continuum of RRMS with joint pathology and symptoms [5]. As such, differential diagnosis of SPMS from RRMS represents a considerable challenge in clinical practice for individuals, especially when they have recently transitioned to the progressive stage. The diagnosis, when it is finally made, often takes several years when severe tissue damage has possibly been done, limiting optimal intervention opportunities. Previously, different machine learning and deep learning methods were proposed to evaluate differing activities of MS. However, most of these studies focused on distinguishing MS from control subjects [9,28]. The several studies that had indeed concentrated on classifying MS subtypes were not directly comparable to our current work. In the context of 3D deep learning, one small study tried to classify primary progressive MS from RRMS using streamlined T1 brain MRI scans [10], unlike the detection of SPMS based on routine clinical MRI applied here. In a larger study of about 300 subjects, while standard clinical brain MRI with different acquisition protocols were investigated, the dataset was complete and their focus was on distinguishing individuals with high or low disability scores from the same cohort of early MS [11].

Based on 3D deep learning of routine clinical brain MRI data, we found the potential of three methods in identifying individuals who had recently converted to SPMS from those with RRMS, where the best 3D VGG19 model achieved the highest validation performance. Both the 3D ResNet152 and 3D DenseNet-121 were recognized techniques for image classification. Their relatively lack of stability in training/validation as seen in loss functions in the present study might be due to the heterogeneous nature of the data, the small sample size, or specific settings of the models, deserving further verification. Nonetheless, our chosen VGG19 model achieved high testing AUCs that were better than a commonly accepted threshold of 0.7 [29], among other competitive testing results. These findings might indicate that conventional clinical brain MRI such as T1, T2, and FLAIR contained valuable feature patterns reflective of SPMS versus RRMS. Through multi-channel volumetric analysis of the features, these 3D deep learning models likely had captured the differentiating information. The promise of conventional brain MRI in deep learning had been repeatedly shown previously in studies of different diseases in addition to MS although 2D models had been mainly used in the literature [30,31].

Another unique component of our study was the inclusion of scans with unavailable sequences, which was not uncommon in clinical settings and which was believed to impact performance of deep learning models [12]. Several reasons might be associated with the unavailability of T1 brain MRI in current MS imaging including: (1) lower desire for gadolinium contrast, which made the pairing pre- and post-contrast T1 less attractive; and (2) pre-contrast T1 was not mandate in many follow-up scans [32]. Our choice of using data augmentation approaches instead of simply exclusion appeared to be feasible. The template-based method was intuitive and easy to implement, but the imaging features in a specific individual could be minimized due to group average. The deep learning-based CycleGAN method was designed to generate subject-specific images [33], yet it might be limited by sample size needed to train the model, and the associated resources and expertise might not be available in every organization. For this study, we used our pre-trained CycleGAN models to minimize the challenges [16]. Through side-by-side comparisons, our results suggested that augmenting scans with images generated from either of the two tested approaches could lead to acceptable model performance, including the use of a simple customized T1 template.

In our joint analysis of brain MRI and clinical data, the best Combined models showed similar or slightly better results than best MRI only models. In general, valuable complementary information would be expected from multi-modal data although the influence of adding clinical variables into imaging-based deep learning in predictive studies of MS was not fully understood [34]. To gain further insight, we explored two variable-curation and three data fusion strategies. As a common practice in the field, continuous variables such as age were often binned into a small number of groups for streamlining purposes, such as five bins used here. These encoded data would then be integrated into one of the post-convolutional layers of an imaging model. In the current study, this approach seemed to have performed well with all testing AUCs over 0.8, regardless of the model layers used in data fusion. In comparison, the other strategy that expanded the same variables to 100 bins to improve ‘weights’ performed most equivalently when they were fused with the second fully connected layer, suggesting the relevance of data curation methods with model settings.

Additional 3D Grad-CAM exploration demonstrated brain regions associated the most with SPMS compared to RRMS. Subsequent ablation studies further suggested that the importance of these brain regions varied. Except the entire hemispheric areas, the importance appeared to be led by frontal lobes and cerebellum, as well as occipital/temporal lobes and then parietal lobe, more from the left than right hemisphere. The reason underlying the relative dominance of left hemispheric regions was unclear, which could be related to hand dominance of the participants which deserved further investigation in future studies. However, the overall regional importance findings seemed to be consistent with evidence underlying disease mechanisms of MS. Previously, tissue injury in frontal lobe was linked to cognitive impairment in MS [35], and temporal lobe with cortical atrophy [36]. Vision dysfunction was one of the most common manifestations of MS [37], which was largely managed by occipital lobes. Other studies also indicated cerebellum being a major site of demyelination in highly disabled individuals with MS [36,38]. Advanced injury in these regions in SPMS could contribute to increased limb weakness (frontal lobe), sensory deficits (parietal lobe), and coordination loss (e.g., cerebellum) [39]. Such SPMS-centric regions could be centers of progressive pathology contributing to future biomarker identifications.

A few limitations were present with this study. First, the sample size was relatively small, and it came from a single center, limiting generalizability. Further, the imaging datasets were acquired with different settings that increased heterogeneity. However, this characteristic of the data might not be entirely undesirable in the context of deep learning as heterogeneity would also expand the variety of samples following correction of data ‘noise’. Further, the images were geometrically augmented during real-time training of the models, which would also help mitigate the sample size limits. Second, a notable number of scans had unavailable T1 MRI sequences as commonly seen in clinical practice. We investigated two candidate image imputation approaches to mitigate, and both showed the feasibility for further validation. Third, the Grad-CAM method had notable constraints. The heatmaps were not entirely precise in delineating anatomical structures, which might have caused artifacts in group-based analysis. Nonetheless, our study focused on Grad-CAM values that mattered the most (≥95%ile) to minimize false positives. Additionally, we included different ablation experiments, which provided additional quantitative information to explain the findings. Previously Grad-CAM was frequently used with success in the field [14,40]. In the future, we plan to confirm our findings including their generalizability using a larger sample size especially from multi-site resources, investigate different modeling and image imputation approaches, and explore different model interpretation strategies such as class saliency visualization [41] and layer-wise relevance propagation [42].

## 5. Conclusions

This study demonstrated the potential of our 3D VGG19 models for identifying individuals who had recently transitioned to SPMS from RRMS using routine brain MRI and clinical datasets. This work entailed multiple innovative points including the investigation of different 3D deep learning frameworks and modeling approaches, the use of routine clinical datasets that were known to be of increased challenge, and the integration of model interpretation approaches such as 3D Grad-CAM, as well as different ablation studies. With further confirmation, this study could serve as a critical step towards establishing a tool for timely diagnosis of SPMS. This in turn would help early appropriate intervention given the availability of over 20 disease modifying therapies in MS and the benefit of early treatment to disease prognosis [43]. Additionally, further verification of our 3D Grad-CAM and ablation findings could help identify the much-needed biomarkers of progressive MS.

## Figures and Tables

**Table 1:** Clinical information of participating groups involved in this study.

Subtype	RRMS	SPMS	Total	*p*-Value
Age at Onset	31.74 ± 9.98	33.7 ± 11.43	32.72 ± 10.74	0.283 ^1^
Age at Scan	44.81 ± 9.91	49.94 ± 11.48	47.37 ± 10.99	0.005 ^1^
Sex (number Female)	61/70	46/70	107/140	0.005 ^2^
EDSS at Onset (IQR)	1.5 (1.0)	2.8 (2.0)	2.0 (1.5)	< 0.001 ^3^
EDSS at Scan (IQR)	1.5 (0.5)	6.0 (0.5)	6.0 (4.0)	<0.0001 ^3^
Disease Duration	13.21 ± 7.36	16.24 ± 8.99	14.72 ± 8.33	0.050 ^3^
Treatment at Scan	7.90 ± 4.43	9.54 ± 4.73	8.72 ± 4.64	0.053 ^1^

Note: Shown are mean ± standard deviation or median (interquartile range—IQR); ^1^ Independent Student’s *t*-test; ^2^ Chi-Square test; and ^3^ Mann–Whitney U test.

**Table 2:** Testing performance (mean ± standard deviation) of 3D classification models based on the best model of 3D VGG19.

Approach		Accuracy	Precision	Recall	F1_Score	ROC_AUC
MRI-Only						
Template		0.74 ± 0.07	0.76 ± 0.06	0.74 ± 0.27	0.72 ± 0.13	0.82 ± 0.08
CycleGAN		0.71 ± 0.08	0.73 ± 0.09	0.68 ± 0.22	0.69 ± 0.11	0.82 ± 0.07
Combined						
100-bins	Flatten	0.65 ± 0.06	0.70 ± 0.11	0.56 ± 0.23	0.60 ± 0.13	0.74 ± 0.06
	FC1	0.69 ± 0.07	0.71 ± 0.09	0.66 ± 0.18	0.67 ± 0.09	0.80 ± 0.06
	FC2	0.73 ± 0.06	0.75 ± 0.11	0.74 ± 0.24	0.72 ± 0.09	0.81 ± 0.06
Five-bins	Flatten	0.73 ± 0.08	0.76 ± 0.13	0.70 ± 0.00	0.73 ± 0.06	0.84 ± 0.08
	FC1	0.73 ± 0.08	0.69 ± 0.09	0.88 ± 0.16	0.76 ± 0.07	0.83 ± 0.06
	FC2	0.72 ± 0.08	0.72 ± 0.10	0.74 ± 0.23	0.71 ± 0.12	0.82 ± 0.08

Note: FC refers to fully connected layer.

## Data Availability

The data presented in this study may be available from the corresponding author upon reasonable request subject to ethics approval.

## Supplementary Materials

The following supporting information can be downloaded at: https://www.mdpi.com/article/10.3390/brainsci16070670/s1, Figure S1: Loss versus epoch over training and validation.
