# Supplementary material for: Three-Dimensional Deep Learning with Routine Brain Magnetic Resonance Imaging and Clinical Data for Identification of Secondary Progressive Multiple Sclerosis
Source: Brain Sci. 2026 Jun 26;16(7):670. doi: 10.3390/brainsci16070670 (PMC13406842; doi:10.3390/brainsci16070670)
Supplement: Supplementary file 1 [file brainsci-16-00670-s001.zip › brainsci-4352824-supplementary.pdf]

Supplementary Figure S1

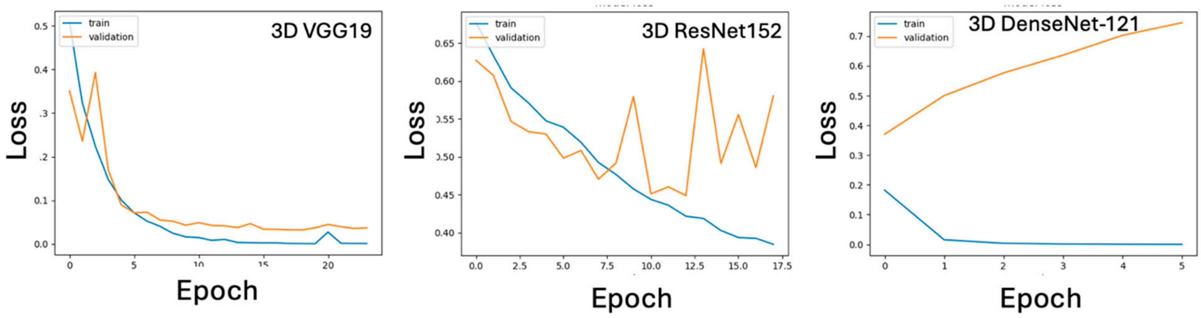

Loss versus epoch over training and validation. Shown are the best model performances from the 3D VGG19, 3D ResNet152, and 3D DenseNet-121 methods, respectively. All models are based on the brain MRI only data.
